# Dual pathogenic variants in *ADAMTS13* and *CFHR1/CFHR3* deletion: divergent thrombotic microangiopathy phenotypes in siblings

**DOI:** 10.1007/s00467-026-07335-1

**Published:** 2026-05-11

**Authors:** Mona Hamed Gehad, Doaa M. Youssef, Amal S. El-Shal, Mona Mohammed Elsharkawy, Amal E. Gohary

**Affiliations:** 1 Department of Pediatrics, Faculty of Medicine, Zagazig University, Al Sharqia Governorate, Zagazig City, Egypt; 2 Medical Biochemistry Department, Faculty of Medicine, Zagazig University, Cairo, Egypt; 3 Medical Biochemistry and Molecular Biology Department, Armed Forces College of Medicine (AFCM), Cairo, Egypt

**Keywords:** Thrombotic microangiopathy, Atypical HUS, *ADAMTS13*, Plasmapheresis

## Abstract

Two male siblings, aged 15 and 13 years, born to consanguineous parents, presented with hereditary anemia and thrombocytopenia associated with unexplained hepatosplenomegaly. Both were initially diagnosed with hereditary cytopenia and maintained on transfusion therapy since childhood. At age 15, the elder sibling developed acute thrombotic microangiopathy (TMA) with hypertensive encephalopathy, hemolytic anemia, severe thrombocytopenia, and acute kidney injury requiring hemodialysis and plasma exchange. The younger sibling exhibited predominantly hematological manifestations with severe anemia and thrombocytopenia but preserved kidney function, responding favorably to plasma transfusion. Whole-exome sequencing identified two distinct pathogenic variants: a homozygous mutation in *ADAMTS13* associated with hereditary thrombotic thrombocytopenic purpura (TTP; OMIM 274150), and a homozygous 57.1 Kb deletion at 1q31.3 involving *CFHR1* and *CFHR3*, associated with atypical hemolytic uremic syndrome (aHUS; OMIM 235400). This case series highlights the phenotypic variability of TMA syndromes and underscores the importance of comprehensive genetic testing in hereditary cytopenia.

## Introduction

Thrombotic microangiopathies (TMAs) are a heterogeneous group of disorders characterized by microangiopathic hemolytic anemia, thrombocytopenia, and organ dysfunction due to microvascular thrombosis. Among these, hereditary thrombotic thrombocytopenic purpura (TTP) results from severe deficiency of *ADAMTS13* activity, while atypical hemolytic uremic syndrome (aHUS) arises from dysregulation of the alternative complement pathway. Genetic variants in *ADAMTS13* and complement regulatory genes, including *CFHR1* and *CFHR3*, are well-established causes of these syndromes [1]. However, the simultaneous occurrence of pathogenic variants in both pathways within a single family is exceptionally rare and may drive diverse clinical phenotypes. We present two siblings with dual genetic predispositions to TMA, illustrating variable disease expression and therapeutic responses.

## Case presentation

We report two male siblings, aged 15 and 13 years, who are the first and third children of a consanguineous marriage. Both presented with hereditary anemia and thrombocytopenia associated with unexplained hepatosplenomegaly. They were initially diagnosed with hereditary cytopenia and had been maintained on transfusion therapy as required since the age of 6 years.

Extensive investigations were performed. Hemoglobin electrophoresis was normal, excluding common hemoglobinopathies. Flow cytometric analysis for paroxysmal nocturnal hemoglobinuria (PNH) demonstrated normal expression of CD55 and CD59. Bone marrow aspirates revealed hypercellular marrow without abnormal or malignant cells. Immunological work-up was unremarkable, with no evidence of immune-mediated cytopenia. Despite these findings, no definitive etiology was established, and the patients continued to receive supportive transfusion therapy on demand.

At 15 years of age, the elder sibling developed sudden pallor, convulsions, and impaired consciousness, prompting admission to our tertiary hospital ICU. Clinical evaluation revealed hypertensive encephalopathy. Laboratory investigations demonstrated features consistent with TMA, including severe thrombocytopenia (platelet count 11 × 10^9^/L), hemolytic anemia (hemoglobin 6 g/dL, reticulocytosis 10%, red cell fragmentation on peripheral smear, elevated lactate dehydrogenase, reduced haptoglobin), and acute kidney injury (serum creatinine 4 mg/dL). Blood pressure was controlled with antihypertensive therapy; however, neurological impairment persisted. Hemodialysis was initiated for uremic encephalopathy, resulting in improvement of consciousness. The patient was subsequently transferred to the nephrology unit for further evaluation and management.

TMA investigations in the elder sibling revealed a factor H level of 72% (reference range, 70–130%); anti-factor H IgG of 4.6 AU/mL (reference ≤ 27 AU/mL); and *ADAMTS13* activity of 66%. Kidney biopsy demonstrated membranous changes, early focal segmental glomerulosclerosis (FSGS) with thrombus formation in a single arteriole. Following therapeutic plasma exchange, the patient showed marked clinical improvement: hemoglobin 12 g/dL, platelets 250 × 10⁹/L, and serum creatinine 1.2 mg/dL.

The younger sibling, aged 13 years, presented with anemia (hemoglobin 7.8 g/dL) and severe thrombocytopenia (platelet count 31 × 10^9^/L) while kidney function was preserved (serum creatinine 0.5 mg/dL). Laboratory evaluation revealed reticulocytosis (10%), elevated lactate dehydrogenase, and normal immunological work-up. TMA studies showed a normal factor H level (101%) and anti-factor H IgG (4.1 AU/mL; reference ≤ 27 AU/mL), but *ADAMTS13* activity was 10%. The patient received plasma transfusion, which led to dramatic improvement, with hemoglobin increasing to 10 g/dL and platelet count normalizing to 210 × 10^9^/L.

Whole-exome sequencing (WES) revealed a dual genetic pathology. First, a homozygous pathogenic frameshift variant was identified in *ADAMTS13* (NM_139027.6:c.2883del: NP_620596.2:p.Cys962AlafsTer3). Absent in gnomAD v4.1.0, it is predicted to cause nonsense-mediated decay and is linked to hereditary TTP (OMIM: 274,150; ClinVar ID: VCV004291762/PMID: 34,306,773). Additionally, a homozygous 57.1 Kb pathogenic deletion at 1q31.3 encompassing *CFHR1* and *CFHR3* [NC_000001.11:g.(?_196774887)_(196831999_?)del (GRCh38)] was detected. While observed in gnomAD SVs v2.1.1, this deletion is a well-established risk allele for atypical HUS (OMIM: 235,400; ClinVar: VCV000005065.1; PMID: 36793547).

## Discussion

TMA in children represents a heterogeneous group of disorders characterized by microangiopathic hemolytic anemia, thrombocytopenia, and variable end-organ involvement, most prominently affecting the kidneys. Advances in molecular diagnostics have revealed that inherited defects in both the *ADAMTS13* pathway and the complement alternative pathway are key drivers of hereditary TMA, yet overlapping genetic abnormalities and variable phenotypic expression remain incompletely understood [2].

The coexistence of a homozygous pathogenic variant in *ADAMTS13* and deletion of *CFHR1* and *CFHR3* represents an exceptionally rare and complex overlap of two distinct TMAs. Both conditions share the final common pathway of microvascular thrombosis and endothelial injury, yet they arise from fundamentally different mechanisms.

We report siblings with identical homozygous *ADAMTS13* and *CFHR1/CFHR3* mutations manifesting divergent phenotypes: the elder with kidney-dominant aHUS and the younger with isolated hematological features of hereditary TTP. This unique “double hit” scenario suggests that disease severity and organ involvement are governed by epigenetic factors or environmental triggers beyond the primary genetic variants.

Previous studies have described similar but distinct conditions. Huang et al. (2025) reported congenital TTP due to compound heterozygous *ADAMTS13* mutations, highlighting the classical hematological phenotype and favorable response to plasma therapy [3]. Separately, Mini et al. (2018) described an infant with aHUS associated with combined partial deficiencies of complement factor H-related proteins and autoantibodies to both factor H and *ADAMTS13*, resulting in a fulminant clinical course [4]. Bitzan et al. (2018) reported acquired TTP in the context of isolated *CFHR1/CFHR3* deletion, with rapid remission following complement blockade, underscoring the potential interplay between complement dysregulation and *ADAMTS13* deficiency [5].

The absence of anti-factor H antibodies in both patients suggests that *CFHR1/CFHR3* deletion may predispose to complement dysregulation through mechanisms independent of autoantibody formation. The phenotypic variability observed underscores the complexity of genotype–phenotype correlations in TMA and highlights the importance of functional assays in addition to genetic testing.

In complex or incompletely explained clinical presentations, particularly within consanguineous families, the discovery of a primary pathogenic variant must not preclude further genomic investigation. Clinicians must maintain a high index of suspicion for dual or oligogenic pathology, utilizing comprehensive WES interpretation to ensure accurate diagnosis, genetic counseling, and targeted therapeutic management.

A key limitation of this study is the absence of targeted parental or extended family segregation testing. While the presence of identical homozygous pathogenic variants in both siblings strongly suggests that each parent is heterozygous for the variants, definitive molecular confirmation is lacking. Testing the biological parents or other family members remains highly recommended to formally confirm variant segregation, which is essential for accurate genetic counseling and familial risk assessment.

## Conclusion

The coexistence of *ADAMTS13* and *CFHR1/3* variants in siblings from a consanguineous pedigree resulted in a wide clinical spectrum of TMA. This phenotypic diversity necessitates a multidisciplinary approach focused on early genetic diagnosis. Such insights are vital for guiding targeted therapies, anticipating complications, and providing accurate prognostic and genetic counseling for affected families.

## Summary

### What is new?

- The unique coexistence of homozygous *ADAMTS13* and *CFHR1/CFHR3* mutations in siblings manifests as divergent clinical phenotypes and therapeutic responses, representing a novel dual-hit TMA.
